# “Sandwich” Strategy to Intensify EGFR Blockade by Concurrent Tyrosine Kinase Inhibitor and Monoclonal Antibody Treatment in Highly Selected Patients

**DOI:** 10.3389/fonc.2022.952939

**Published:** 2022-07-08

**Authors:** Guoqing Zhang, Beibei Yan, Yanan Guo, Hang Yang, Jindong Li

**Affiliations:** Department of Thoracic Surgery and Lung Transplantation, First Affiliated Hospital of Zhengzhou University, Zhengzhou, China

**Keywords:** epidermal growth factor receptor, tyrosine kinase inhibitor, uncommon mutation, NSCLC, drug resistance

## Abstract

*EGFR* TKIs are not curative, and targeted resistance inevitably results in therapeutic failure. Additionally, there are numerous uncommon *EGFR* mutations that are insensitive to *EGFR* TKIs, and there is a lack of clinical strategies to overcome these limitations. EGFR TKI and mAbs target EGFR at different sites, and a combination regimen for delaying/preventing resistance to targeted therapy or obtaining more intensive inhibition for uncommon mutations at cellular, animal and human levels has been explored. This review critically focuses on a combination strategy for uncommon *EGFR* mutation-positive NSCLC, and discuss the preclinical data, clinical implications, limitations and future prospects of the combination strategy.

## Introduction

Over the past decades, systemic treatment strategies involving epidermal growth factor receptor (*EGFR*) tyrosine kinase inhibitors (TKIs) have greatly improved outcomes and represent the backbone of treatment for advanced *EGFR*-mutant non-small-cell lung cancer (NSCLC) (1, 2). *EGFR* TKIs are now the standard treatment for classic mutations (such as exon 19del and exon 21 L858R point mutations), with median progression-free survival (PFS) ranging from 10-12 months (2–4) and 18.9 months (5) for first/second-generation *EGFR* TKIs and third-generation TKIs, respectively. However, *EGFR* TKIs are not curative, and targeted resistance inevitably results in therapeutic failure (6). Moreover, various primary and acquired uncommon mutations (such as *EGFR*-dependent or *EGFR*-independent mutations) have been reported, and such uncommon *EGFR* mutations may be associated with poor response (7) or even resistance (8) to *EGFR*-TKI monotherapy.

Comprehensive next-generation sequencing (NGS) has greatly promoted clinical research on targeted therapy. Strategies for targeted therapy to delay drug resistance or find effective targeted strategies for uncommon mutations have always been a hot topic in clinical research. Despite available information on resistance mechanisms and uncommon mutations, there is a paucity of clinical strategies for overcoming these limitations. To date, *EGFR* TKI combination therapy [with cytotoxic anticancer agents (9–12), angiogenesis inhibitors (13–16), or TKIs (17)] have been widely explored in this heterogeneous group of patients with regard to efficacy, safety and tolerability. Studies have also begun exploring combination therapy with *EGFR* TKIs and mAbs; we call this the “sandwich” strategy because EGFR is blocked by integrating *EGFR* TKIs intracellularly and *EGFR* mAbs extracellularly) (18, 19).

This review focuses specifically on the “sandwich” strategy for *EGFR* mutation-positive NSCLC, aiming to overcome drug resistance and discuss prospects for their use in clinical settings.

## Mechanisms of Limited Responsiveness to EGFR-TKIs With Uncommon *EGFR* Mutations

Under normal physiological processes, EGFR forms a dimer when bound by ligands, such as EGF, EPF, TGFα, AR, BTC, HB-EGF and EPR, after which autophosphorylation of the tyrosine kinase domain (TKD) occurs, transmitting pro-proliferation signals in cells (20). Under circumstances of *EGFR* driver mutation, the TKD is homeostatically activated in a ligand-independent manner, leading to transmission of excessive pro-survival and pro-proliferation signals and resulting in cancer initiation and progression (21). The efficacy of EGFR mAbs involves blocking the binding of ligands to EGFR (part of the mechanism), inhibiting ligand-induced activation of TKD (22); the efficacy of EGFR TKIs is related to TKD binding, decreasing the relative affinity of TKD for ATP in a ligand-independent manner (23).

**Figure 1:**
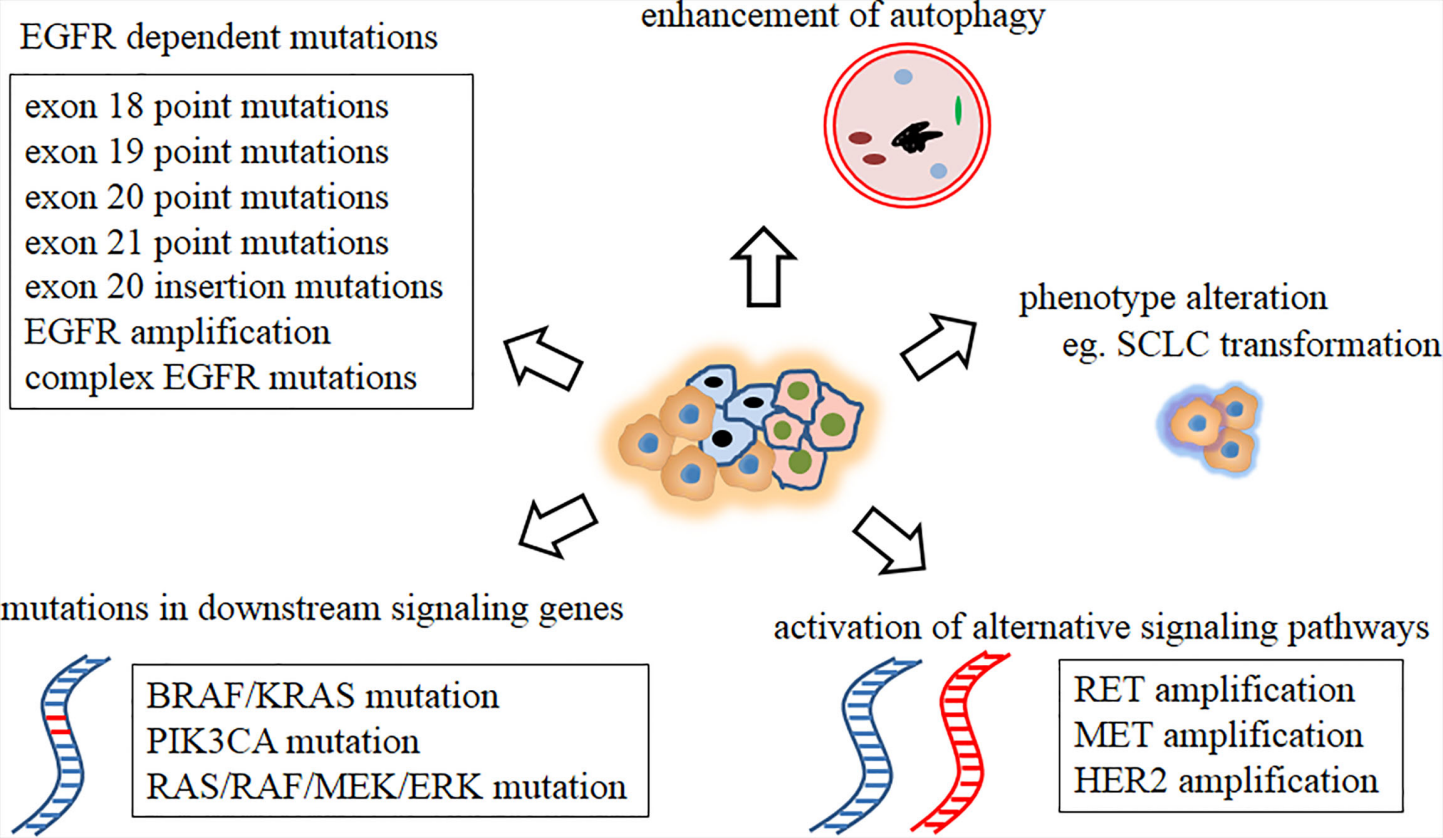
Mechanisms of drug resistance to EGFR TKIs. EGFR, epidermal growth factor receptor; SCLC, small cell lung cancer; TKI, tyrosine kinase inhibitor.

Intratumor heterogeneity (ITH) (24, 25) is defined as the a tumor containing different tumor cells (TCs) with different genomic features. Several studies (26, 27) have described the ITH and evolutionary process of NSCLC. Primary or acquired resistance is a direct consequence of preexisting ITH and continuous development of new therapy-resistant phenotypes. Broadly five mechanisms of drug resistance (primary and acquired) to EGFR TKIs have been reported, as follows (8): 1. EGFR-dependent mutations (5) (including exon 18 point mutation (L718Q, G724S), exon 19 point mutation (D761Y, L747S/P), exon 20 point mutation (S768I, T790M, L792F/H, G796S/R/D, C797S), exon 21 point mutation (V843I, T854A), exon 20ins mutation (28) and *EGFR* amplification (8, 29), with some complex *EGFR* mutations reported to be responsible for resistance acquisition (8, 30)); 2. *EGFR*-independent mutations (8), such as activation of alternative signaling pathways, including RET amplification, MET amplification, and HER2 amplification; 3. Mutations in downstream signaling genes (31) such as BRAF/KRAS mutation, PIK3CA mutation, and RAS/RAF/MEK/ERK mutation; 4. phenotype alteration, such as small-cell lung cancer (SCLC) transformation (29); 5. enhancement of autophagy (6) (**Figure 1**). Furthermore, these mechanisms may be coactivated in a single case, and their crosstalk can further complicate treatment and worsen patient outcome.

Targeting kinases with small molecular TKI directed at their well-characterized ATP-binding pockets or allosteric pockets has been carried out (32). Structures of uncommon mutations may interfere with the binding of targeted drugs to ATP-binding pockets or allosteric pockets, which may be a reason for the limited responsiveness to EGFR TKIs. Cases of uncommon *EGFR* mutations are highly heterogeneous (33). Specifically, these uncommon mutations consist of gatekeeper mutation (T790M), mutation causing steric hindrance (L718Q, L844V), mutation modifying the TKI-binding site (L798I, C797S), solvent-front mutation (G796S/R/D) and mutation within the hinge region (L792F/H). The T790M mutation in *EGFR* is located at a special position; it is often referred to as the “gatekeeper residue”, a structural location documented to interfere with inhibitor binding (20). By *in silico* protein structure modeling for TKI binding, Yang and colleagues revealed that the L718Q and L792H substitutions prevent osimertinib (a third-generation EGFR TKI) binding by introducing spatial confliction and decreasing local hydrophobicity. Furthermore, the L792 and L718 mutations markedly increase the half inhibitory concentration (IC50) of osimertinib *in vitro (* 31), consistent with the *in vivo* conclusion. L844V mutation is reported to reduce WZ4002 (a third-generation EGFR TKI) binding and alter hydrophobic contacts with its inhibitor (34). As the second-generation irreversible EGFR TKI afatinib/dacomitinib and the third-generation EGFR TKI osimertinib bind covalently to Cys797 in the ATP-binding pocket (35), the occurrence of secondary mutations near the binding site (C797S (36), L798I (37)) theoretically leads to drug resistance. Uchibori *et al.* proved that C797S mutations reduce the affinity between osimertinib and the EGFR kinase domain and increase the relative affinity for ATP (38).

## Possibility of the “Sandwich” Strategy in NSCLC

Using a highly sensitive locked nucleic acid (LNA)-based method, T790M has been detected in up to 68% of cases of EGFR TKI acquired resistance (39). Regarding drug resistance mechanisms to TKIs, *EGFR*-dependent mutations are observed in most cases (T790M for first/second-generation and loss of T790M and other *EGFR* uncommon and complex mutations for third-generation) (29). These data suggest instability of the *EGFR* signaling pathway or insufficient inhibition of EGFR by TKIs. Thus, it may be reasonable to combine EGFR TKI and EGFR mAbs for more intensive consolidation therapy in selected patients. Currently, EGFR TKIs used for the “sandwich” strategy include gefitinib, erlotinib, afatinib, EAI045, brigatinib and lazertinib; *EGFR* mAbs include cetuximab, necitumumab, panitumumab and amivantamab.

**Figure 2:**
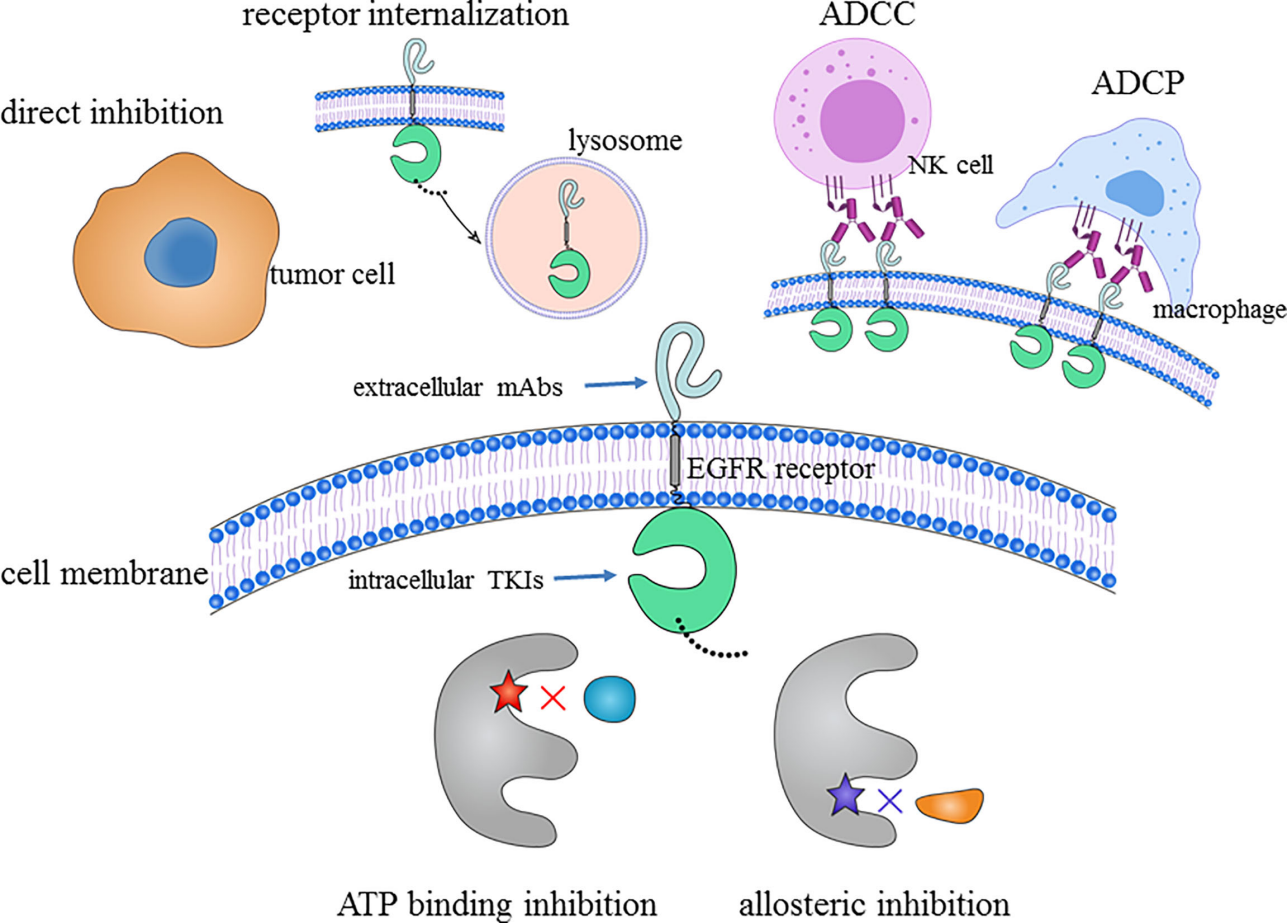
Antitumor mechanisms of the “sandwich” strategy: extracellular domain targeted by EGFR mAbs and intracellular domain targeted by EGFR TKIs. ADCC, antibody-dependent cellular cytotoxicity; ADCP, antibody-dependent cellular phagocytosis; EGFR, epidermal growth factor receptor; mAb, monoclonal antibody; NK, natural killer; TKI, tyrosine kinase inhibitor.

EGFR TKI and EGFR mAbs both target EGFR; however, some of their mechanisms of action and their blocking effects do not completely overlap (**Figure 2**). Low-molecular-weight TKIs block EGFR signaling by either competing with ATP (20) or changing the structure of EGFR (known as allosteric inhibition) (40). For high-molecular-weight mAbs, EGFR mAbs have the following mechanisms in addition to direct tumor inhibition by preventing ligand binding by blocking the ligand-binding extracellular domain. Under normal biological processes, the binding of ligand to EGFR results in cell surface receptor number downregulation *via* internalization of the ligand–receptor complex, which is eventually degraded in lysosomes (41). EGFR mAbs have the capacity to form receptor-containing complexes, which attenuate *EGFR* pathway signaling through receptor internalization. Additionally, studies have shown that EGFR TKIs can induce *EGFR* upregulation (8, 42–44), and EGFR mAbs have been proven to be able to decrease the level of cell surface *EGFR (* 38) and abrogate the increase in TKI-induced *EGFR* transcription (44). From an immunological perspective, mAbs (such as cetuximab and necitumumab) of the IgG1 subtype have the ability to preferentially enhance affinity toward binding Fc with FcγR, leading to antibody-dependent cellular cytotoxicity (ADCC) or antibody-dependent cellular phagocytosis (ADCP) of tumor cells (45, 46). Interestingly, the human IgG2 isotype EGFR antibody panitumumab has Fc-mediated effector functions (45, 47). The mechanistic explanation is that panitumumab is highly potent in recruiting myeloid effector cells (such as PMN and M1 macrophages) for tumor cell killing by ADCC and ADCP (48). Therefore, theoretically, EGFR TKIs and mAbs exert synergistic antitumor effects while lowering the dose required for efficacy.

EGFR TKI and EGFR mAb target *EGFR* at different sites, and preclinical and clinical studies have revealed shared and complementary mechanisms of action, resulting in superior inhibition of EGFR, MAPK (mitogen-activated protein kinase), Akt phosphorylation, induction of apoptosis and vascularization inhibition of xenografts (49). The “sandwich” strategy has been explored in delaying/preventing resistance to targeted therapy or obtaining more intensive inhibition for uncommon mutations at the cellular, animal and human levels.`

### Preclinical Data of the “Sandwich” Strategy

Early in the last century, scientists realized the unique capability of this combination regimen for EGFR inhibition (50). Numerous studies have demonstrated the benefit of EGFR TKI plus EGFR mAbs in lung cancer using *in vitro* and *in vivo* model systems (**Table 1**). Huang et al. found that gefitinib/erlotinib plus cetuximab shows significant cell proliferation inhibition, apoptosis promotion and tumor suppression in lung cancer cell lines and human lung cancer xenografts (51).

**Table 1:** Preclinical data evaluating the “sandwich” strategy.

Year and author	Country	Strategy	Study object	Treatment response
1997 Bos (50)	USA	PD153035+cetuximab	Cell lines overexpressing EGFR	cell proliferation
2004 Matar (49)	Spain	Gefitinib+cetuximab	Vulvar squamous carcinoma cell line (A431), colon carcinoma cell line (DiFi), prostate carcinoma cell line (DU145), and breast carcinoma cell lines (SK-BR-3, MDA-MB-435S, MDA-MB-468, MDA-MB-453, and T-47D,BT-474) A431 xenografts	cell proliferation tumor regressions
2004 Huang (51)	USA	Gefitinib/Erlotinib+cetuximab	Lung/head/neck cancer cell lines human lung cancer xenografts	cell proliferation/apoptosis tumor regressions
2005 Jimeno (44)	USA	Erlotinib+cetuximab	HuCCT1 cell lines A431, HuCCT1, and Panc430 xenografts	cell proliferation/apoptosis tumor regressions
2009 Lucia Regales (52)	France	Gefitinib/erlotinib+cetuximab/panitumumab	EGFR-expressing TNBC cell lines	
2016 EI Guerrab (53)	Japan	Afatinib+ cetuximab	Ba/F3 cell lines (A763_Y764insFQEA, Y764_V765insHH, A767_V769dupASV, D770_N771insNPG) xenografts (EGFR A767_V769dupASV or Y764_V765insHH).	cell proliferation tumor regressions
2016 Pirazzoli (54)	USA	Afatinib+ cetuximab	xenografts (EGFR L858R+T790M)	tumor regressions
2016 Jia (55)	USA	Afatinib+ cetuximab	xenografts (EGFR L858R)	time to relapse and incidence of drug-resistant tumors
2017 Uchibori (38)	USA	EAI045+cetuximab	Genetically engineered mouse (EGFR L858R/T790M or EGFR L858R/T790M/C797S)	tumor regressions
2018 Della Corte (56)	Japan	Brigatinib+ cetuximab/panitumumab	EGFR-triple-19del expressing PC9 lung cancer cell lines and xenografts	cell proliferation tumor regressions and survival periods
2019 Hasegawa (57)	Italy	Osimertinib+cetuximab	HCC827 (E746-A759del/T790M), H1975 (L858R/T790M), PC9-T790M (E746-A759del/T790M) cell lines and xenografts	tumor regressions and survival periods
2021 Corso (58)	Italy	erlotinib+cetuximab	EGFR-amplified gastroesophageal adenocarcinoma PDX model	tumor regressions

*EGFR, epidermal growth factor receptor; PDX, patient-derived xenograft model; TNBC, triple-negative breast cancer.

Subsequent studies began to focus on specific types of mutations. *EGFR* exon 20ins is reportedly resistant to EGFR TKIs, and the afatinib- or osimertinib-based “sandwich strategy” has been proven to induce a more potent inhibitory effect against several *EGFR* exon 20ins mutations than either therapy alone in Ba/F3 cells (A763_Y764insFQEA, Y764_V765insHH, A767_V769dupASV, and D770_N771insNPG) and BALB/c-nu mice (*EGFR* A767_V769dupASV and EGFR Y764_V765insHH) (57). A similar study evaluated combinations of osimertinib + cetuximab in several NSCLC models with certain complex mutations (E746-A759del/T790M, L858R/T790M, and E746-A759del/T790M) as second-line treatment following development of resistance to osimertinib, and the results demonstrated that osimertinib + cetuximab may be a novel effective therapeutic option (56) Nevertheless, targeted treatment for patients with *cis*-C797S/T790M/classic mutations is difficult, and no effective therapeutic strategies have been reported. Uchibori et al. found brigatinib to be effective against *cis*-C797S/T790M/classic mutations *in vitro* and *in vivo*, and its effect was markedly enhanced by combination with cetuximab/panitumumab (38). Allosteric inhibitors also play an important role in the “sandwich” strategy. For example, EAI045 is an allosteric inhibitor targeting drug-resistant EGFR mutants with high selectivity and simultaneously spares wild-type EGFR. Jia et al. (55) tested the *in vitro* and *in vivo* efficacy of EAI045 in genetically engineered Ba/F3 cells and a mouse model, and only EAI045 in combination with cetuximab was effective for NSCLC driven by *EGFR* L858R/T790M and L858R/T790M/C797S.

These preclinical studies suggest a synergistic effect between EGFR TKIs and EGFR mAbs. However, it is not yet clear whether we can translate this research evidence from the preclinical stage to humans.

### Clinical Implications of the “Sandwich” Strategy

A study evaluated the efficacy of gefitinib combined with cetuximab for patients with advanced/metastatic NSCLC previously treated with platinum-based chemotherapy without *EGFR* mutation or amplification, and the results showed that none achieved partial response (PR) (59). A similar result was obtained in a study evaluating erlotinib combined with cetuximab for patients with acquired resistance to erlotinib (ORR=0%) (60). Interestingly, the “sandwich strategy” based on afatinib exhibited significant activity, with an ORR of 29% and a median PFS of 4.7 months in advanced *EGFR*-mutant NSCLC and acquired resistance to erlotinib/gefitinib. Furthermore, the results showed comparable treatment effects in patients with T790M-postive or -negative tumors (60). Inspired by the afatinib-based “sandwich strategy”, the randomized phase II, multicenter trial SWOG S1403 evaluated the benefit of afatinib plus cetuximab compared with afatinib alone as first-line treatment targeting classic mutations (exon 19del and exon 21 L858R). Unfortunately, the results showed that the addition of cetuximab to afatinib did not improve outcomes, with 30% of patients in the combination regimen discontinuing cetuximab due to intolerable adverse events (AEs) (61). As an EGFR-MET bispecific antibody, amivantamab (approved by the FDA in 2021) has also been explored in combination with lazertinib in cases progressing on osimertinib (CHRYSALIS), and encouraging preliminary antitumor activity was observed, with an ORR of 36% (62, 63). CHRYSALIS-2 (64) is an ongoing phase 1/1b open-label study evaluating the effects of amivantamab + lazertinib in patients with *EGFR*-mutant NSCLC (first-line therapy) and a phase 3 study (MARIPOSA) in ongoing to assess activity of the combination in *EGFR*-mutant NSCLC (first-line therapy) (65).

Van Veggel *et al.* reported four stage IV NSCLC patients with *EGFR* exon 20ins treated with afatinib (40 mg/d) and cetuximab (250-500 mg, q2w); 3/4 patients (Ser768_Asp770dup, Asn771_His773dup and Ala767_Val769dup) showed a partial response (PR), whereas the remaining (His773dup) showed stable disease (SD); the mPFS was 5.4 months (66). Osimertinib (80 mg/d) plus cetuximab (400 mg, q2w) is also effective for the complex mutation *EGFR* A767_S768insSVD and EGFRex20-ins, and the authors concluded that high-dose osimertinib (160 mg/d) can be considered for such patients, with good tolerance (67).

C797S and T790M mutations may occur either in *cis* or *trans* in NSCLC (68). In general, tumors harboring the *trans*-C797S/T790M/classic mutation are sensitive to a combination of first- and third-generation TKIs (69–71), and *cis*-C797S/T790M/classic mutations are resistant to first/third- and first plus third-generation EGFR TKIs (36, 72). Preclinical studies have demonstrated brigatinib plus cetuximab to be effective for tumors with *cis*-C797S/T790M/classic mutations *in vitro* and *in vivo (* 38). Wang et al. reported the first clinical evidence for the combination of brigatinib (90 mg/d) and cetuximab (600 mg, q4w) against the *cis*-C797S/T790M/19del mutation, with a remarkable PFS of 9 months (36). Interestingly, *trans*-C797S/T790M/classic mutations treated with a combination of first- and third-generation TKIs resulted in rapid drug resistance [PFS was 1 month in a case reported by Arulananda (71)] or evolved to *cis*-C797S/T790M/classic mutations in a short time (69, 70). Zhou et al. found that addition of bevacizumab to osimertinib and brigatinib may delay drug resistance (69). Overall, further research is needed to assess whether it is reasonable to apply brigatinib and cetuximab early before drug resistance occurs under such circumstances.

For some rare complex *EGFR* mutations, EGFR TKIs plus EGFR mAbs are still therapeutically effective. We have reported a rare case of lung adenocarcinoma with a novel *EGFR*–intergenic region (*IGR*) (*SEC61G*) fusion (the mutation frequency was 79.8%) and *EGFR* amplification (copy number: 5.6). The patients received first-line targeted combination therapy with gefitinib (250 mg/d) and cetuximab (500 mg/m2, q2w) and achieved PR according to RECIST guidelines (19).

Interestingly, several preclinical trials of combinations of various EGFR TKIs and EGFR mAb targeting EGFR were successful but failed to translate into clinically significant results (59, 61, 73, 74) (**Table 2**). There are several reasons for such inconsistencies when comparing preclinical and clinical studies.

**Table 2:** Clinical data evaluating the “sandwich” strategy.

Year and author	Country	Phase	Strategy	Study object	ORR	SD	PD	PFS	≥3 AE
2008 Ramalingam (59)	USA	I N=13	Gefitinib: 250 mg/d, cetuximab:100, 200, or 250 mg/m2,qw	prior platinum-based chemotherapy	0%	31%	62%	NA	31%(250 mg/m2)
2011 Janjigian (73)	USA	I/II N=19	Erlotinib: 100 mg/d cetuximab: 250 mg/m2, 375 mg/m2, and 500 mg/m2),q2w	Acquired resistance to erlotinib	0%	89.5%	10.5%	NA	NA
2014 Janjigian (75)	USA	Ib N=126	Afatinib* Cetuximab: 500 mg/m2,q2w	advanced EGFR-mutant lung cancer(acquired resistance to erlotinib/gefitinib)	29%	41.3%	21.4%	4.7 m	46%
			Brigatinib+ cetuximab						
2017 Horn (74)	USA	Ib N=36	Afatinib: 40 mg/d Cetuximab:250-500 mg/m2,q2w	Progression on afatinib	11.1%	38.9%	30.6%	2.9 m	NA
2018 van Veggel (66)	Netherlands	Case N=4	Afatinib: 40 mg/d Cetuximab:250-500 mg/m2,q2w	Ser768_Asp770dup, Asn771_His773dup, Ala767_Val769dup, His773dup	75%	25%	NA	4.5 m	25%
2019 Wang (36)	China	Case N=1	Brigatinib: 90 mg/d Cetuximab: 600 mg, q4w	cis-C797S/T790M/19 del	100%	NA	NA	9 m	No
2019 Fang (67)	China	Case N=1	Osimertinib: 80➔160 mg/d cetuximab: 400 mg, q2w	EGFR A767_S768insSVD and EGFRex20-ins.	100%	NA	NA	>5 m	No
2020 Goldberg (61)	USA	II N=168	Cetuximab+ Afatinib*	exon 19del and exon 21 L858R	67%	NA	NA	11.9	70%
			Afatinib*	exon 19del and exon 21 L858R	74%	NA	NA	13.4	40%
2020 Cho (63)	Korea	I N=23(ongoing)	amivantamab+lazertinib	Progressing on osimertinib	43.5%	39.1%	NA	NA	7%
2021 Joshua (62)	Korea	I N=45(ongoing)	amivantamab+lazertinib	Progressing on osimertinib	36%	NA	NA	4.9 m	NA
2021 Zhang (19)	China	Case N=1	Gefitinib: 250 mg/d Cetuximab: 500 mg/m2,q2w	EGFR-IGR fusion and EGFR amplification	100%	NA	NA	>2 m	No

AE, adverse event; EGFR, epidermal growth factor receptor; NA: not applicable; ORR, objective response rate; PD, progressive disease; PFS, progression-free survival; SD, stable disease; TNBC, triple-negative breast cancer.

* Afatinib 40 mg orally daily, cetuximab 500 mg/m2 intravenously every 2 weeks.

First, studies with significant results usually included specific mutation sites, either preclinical or clinical. That is, this combination is effective against only specific uncommon *EGFR* mutations. For example, although EAI045 in combination with cetuximab can dramatically inhibit tumors harboring *EGFR* L858R/T79M/C797S in mice, this combination does not well target 19del/T79M/C797S (55). Second, there is limited drug dosage in humans. For example, afatinib and dacomitinib are effective at inhibiting *EGFR* T790M *in vitro* and in preclinical models, but their clinical utility for *EGFR* T790M is hampered because the clinical doses required to effectively inhibit this mutation *in vivo* are high and cause severe toxicity (35). In general, efficacy is significantly related to dosage: high doses of EGFR TKIs and mAb result in optimal regression of large tumors in animals (49), but high dosages of drugs usually lead to complicated toxicity situations in humans (59). Third, a more complex tumor microenvironment exists in the human body, and no animal model is a complete replica for a process within humans (76). Indeed, one study showed that only approximately 1/3 of animal research translates into successful human research (77). Fourth, when we compare preclinical and clinical studies, mutation in the subjects included (if any) may have a critical difference in the success or failure of the experiments/clinical trials. A “sandwich” strategy that achieves more precise intervention (*EGFR* mutation before treatment) will result in better disease control (75). An erlotinib/afatinib-based “sandwich” strategy for patients with acquired resistance to erlotinib (73)/afatinib (74) has significantly reduced effectiveness. When we compare SWOG S1403 (first line: treatment naïve mutation) (61) with the study by Janjigian (second line: acquired resistance) (75), we conclude the existence of differences in the biology of untreated disease compared with that of acquired resistance (uncommon mutations may be the targeted beneficiary group by the “sandwich” strategy). In terms of medication, the second-generation irreversible pantarget inhibitor afatinib may confer more complete inhibition of ErbB family members. Cetuximab was used at a higher dose (500 mg/m^2^) in the afatinib-based “sandwich” strategy (59, 73, 75) (**Table 1**). Fifth, negative experimental results are less likely to be published (78), which may create the illusion of success for all published preclinical studies.

## “Sandwich” Strategy for Other Tumors

In addition to NSCLC, several malignancies, such as glioblastoma multiforme, glioblastoma, breast cancer, vulvar squamous carcinoma, prostate carcinoma, head and neck cancer, and gastrointestinal cancers (stomach, colorectal, and pancreatic carcinomas), are associated with EGFR mutation or amplification. Therefore, EGFR-targeted therapy should theoretically be effective.

As early as 2004, Matar et al. explored the blocking effect of gefitinib and cetuximab on the *EGFR* pathway using human cancer cell lines (vulvar squamous carcinoma, prostate carcinoma cells and breast carcinoma cells) and A431 (vulvar squamous carcinoma) xenografts in nude mice (49). The results demonstrated that the “sandwich” strategy resulted in a synergistic effect with regard to the inhibition of cell proliferation and regression of tumors by targeting EGFR (49). Similar conclusions were reported with the gefitinib/erlotinib and cetuximab combination for head/neck cancer (51). Triple-negative breast cancer (TNBC) is characterized by poor prognosis, and a study suggested that the “sandwich” strategy might result in an enhanced antitumor effect *in vitro* (53).

With regard to the gastrointestinal digestive system, colorectal cancer escapes *EGFR* blockade by downstream signaling activation (RAS-MEK), and combination of *EGFR* blockade and MEK blockade can prevent resistance both *in vitro* and *in vivo (* 79). Additionally, gefitinib and cetuximab can inhibit colon carcinoma cell proliferation and promote tumor regression in nude mice (49). *EGFR* amplification predicts aggressive biological behavior and poor prognosis. Preclinical trials performed on PDX models with *EGFR*-amplified gastroesophageal adenocarcinoma revealed that the cetuximab and lapatinib/erlotinib combination still results in a deep and durable response (58). Similarly, in a study evaluating the biliary tract cancer cell line HuCCT1 (*in vitro* cell proliferation inhibition and apoptosis promotion) and pancreatic cancer cell line Panc430 xenografts, tumor growth *in vivo* was significantly decreased with combined therapy (gefitinib/erlotinib and cetuximab) (44).

However, the data about the “sandwich” strategy are fairly limited in clinical settings. Most of the studies have focused on EGFR TKIs or EGFR mAbs, rather than their combination. A randomized phase 2 study (NCT01919879) comparing cetuximab and cetuximab plus afatinib in refractory wtKRAS metastatic colorectal cancer has been completed, but the results have not yet been reported (80).

## Limitations and Prospects of the “Sandwich” Strategy

The “sandwich” strategy exerts a stronger antitumor effect than a single drug and has certain clinical prospects for NSCLC, especially in patients with certain kinds of uncommon *EGFR* mutations. However, this combination is far from optimal, which limits its wide clinical use.

### Relatively Severe Toxicity of the “Sandwich” Strategy

The ultimate goal of targeted therapy is low toxicity and high efficiency. The most prominent problem of the “sandwich” strategy is its relatively increased inhibition of wild-type EGFR, leading to intolerable toxicity. Based on clinical data for patients with acquired resistance to first-generation TKIs, therapy-related grade ≥3 adverse events of the afatinib-based “sandwich” strategy occurred in 46%; 36% required a dose reduction, and 13% discontinued therapy due to intolerable AEs (75). Based on SWOG S1403, therapy-related grade ≥3 AEs with afatinib plus cetuximab and afatinib occurred in 70% and 40% of patients, respectively, and a dose reduction of afatinib to 30 mg occurred in 56.7% and 26.2% of patients, respectively; 30% discontinued cetuximab therapy due to intolerable AEs in the combination group (61). It is noteworthy that the first/third-generation TKI-based “sandwich” strategy appears to have good tolerance, with only 31% reporting ≥3 AEs (59). However, the sample size for this combination is relatively small, involving mostly case reports (19, 59, 67, 73). Considering the rate of grade ≥3 AEs with TKI monotherapy (gefitinib 31% (81), erlotinib 45% (2), afatinib 36-40% (4), osimertinib 34% (5)), we conclude that AEs of the first/third-generation TKI-based “sandwich” strategy may be clinically acceptable but that the afatinib-based “sandwich” strategy significantly increases the occurrence of AEs compared to monotherapy. Thus, considering the various options for patients harboring common *EGFR* mutations as well as T790M mutations, the “sandwich” strategy may not be a priority selection regarding effectiveness and safety. Recent studies have shown that the novel “sandwich” strategy, amivantamab+lazertinib, may significantly reduce AEs, with grade ≥3 AEs occurring in only 7% of patients. Therefore, novel, more effective and safer therapeutic strategies may provide new insight into the “sandwich” strategy.

### Drug Resistance of the “Sandwich” Strategy

Resistance to targeted therapy is inevitable, and “sandwich” strategies are no exception. In the era of precision targeted therapy, gene mutations should be rechecked after drug resistance to guide subsequent treatment. Due to limited clinical application, the mechanism of drug resistance of the “sandwich” strategy is far from understood, and it seems that *EGFR*-independent mutations will be the majority because of sufficient inhibition of EGFR. However, Pirazzoli et al. proved that an afatinib-based “sandwich” strategy does not suppress emergence of T790M *KRAS* mutations (such as G12R, G12V and G12D) and that *EGFR* amplification is associated with resistance to an afatinib-based “sandwich” strategy (54). Additionally, mutations in downstream signaling genes (*RAS/RAF/MEK/ERK, PI3K/AKT/mTOR*) are induced (54). Combined targeting with three drugs (osimertinib, bevacizumab and brigatinib) in patients with *cis*-C797S/T790 M/L858R mutation seems to delay the time to incidence of drug resistance (72). Preclinical data suggest that the afatinib-based “sandwich” strategy markedly delays drug resistance in transgenic mice harboring *EGFR* L858R (54); however, clinical studies have not yielded similar results. Inspired by a study by Zhao et al. (72), introduction of vascular endothelial growth factor receptor (VEGFR) TKIs may modify the resistance mechanism, which requires further study.

### Future Prospects of Targeted Therapy

Historically, reversal of drug resistance to first-generation TKIs (with T790M as the most common resistance mutation) has been explored by implementing the “sandwich” strategy (73, 75) because there are no better treatment options. However, with the development of targeted therapies, a large number of targeted drugs (such as osimertinib (82), almonertinib (83), avitinib (84), and furmonertinib (85)) have been developed with high selectivity for the mutant type, which further reduces the value of such a strategy for this kind of mutation. for the C797S mutation, brigatinib has been shown to be effective at inhibiting *cis*-C797S/T790M/del19 but spares wild-type cells (38). Another novel highly potent selective 4th-generation *EGFR* TKI BLU-945 also has promising activity for the treatment of *EGFR* T790M/C797S resistant NSCLC, with high selectivity, and the ongoing SYMPHONY will reveal more data (86). The novel *EGFR* TKI mobocertinib (87) and third-generation TKI furmonertinib (approval in China in March 2021 (88)) (89, 90) have been shown to be effective for *EGFR* exon 20ins disease. Studies have shown that the effect of treatment with immunotherapy in patients harboring *EGFR* exon 20ins mutations is poor (91) and can even lead to hyperprogressive disease (92). Interestingly, the EGFR–MET-targeted bispecific antibody amivantamab (approved by the FDA in May 2021) yielded robust and durable responses to *EGFR* exon 20ins-mutated tumors after progression on platinum-based chemotherapy (74, 93).

Numerous nonsensitive mutations exist, and combination treatment may drastically reduce the number of remaining tumor cells compared to a single drug. Combination drugs often indicate combined AEs. Theoretically, a single agent targeting nonsensitive mutations may be of great significance, similar to bispecific antibodies in the field of cancer immunotherapy, such as amivantamab (31, 34).

What is the potential for the “sandwich” strategy? First, as we described, *EGFR* TKI and *EGFR* mAbs may exert synergistic antitumor effects, suggesting the possibility that we can reduce the dose of a drug to control AEs without impairing the therapeutic effect. Second, with the precision of medical interventions, treatment for tumors with specific uncommon *EGFR* mutations will undoubtedly be tailored more individually, especially with the “sandwich” strategy (relatively severe toxicity). Third, research and development of new drugs with high efficacy and low toxicity may offer breakthroughs in the “sandwich” strategy. A novel combination amivantamab + lazertinib is being explored as a first-line or multiple-line treatment, with promising therapeutic effects.

As another cornerstone of therapy, the role of chemotherapy in NSCLC should not be ignored. Nonetheless, studies have shown that TKIs are not inferior to or even better than chemotherapy with respect to disease control in patients with uncommon *EGFR* mutations (such as exon 20ins, L861Q and G719X) (94, 95). Studies have also demonstrated that chemotherapy for patients with uncommon mutations may result in significantly better survival (96, 97) than with EGFR-TKIs, despite worse mPFS (97). Therefore, it is currently unknown how to best sequence these different therapies. There is also a lack of evidence with respect to the advantages and disadvantages of chemotherapy and the “sandwich” strategy. Further studies are needed to investigate appropriate drug sequences.

## Conclusions

In this review, we focus on several EGFR-dependent mutations for which a “sandwich” strategy is possible. Our review highlights that dual EGFR inhibition is particularly meaningful in this patient population. We conclude that the “sandwich” strategy may have limited benefit in patients with previously untargeted classic *EGFR* mutations and is expected to improve the prognosis of NSCLC patients harboring certain uncommon *EGFR* mutations. However, their relatively severe toxicity limits their clinical application, and there is an urgent need to develop new targeted drugs with less inhibition of wild-type EGFR.

## Author Contributions

GZ, BY, and YG contributed to conception and design of the study. GZ, BY, YG and HY organized the database. HY performed the statistical analysis. GZ wrote the first draft of the manuscript. All authors contributed to manuscript revision, read, and approved the submitted version.

## Funding

This study was supported by the Key Scientific Research Projects of Institutions of Higher Learning in Henan Province (No. 21A320032).

## Conflict of Interest

The authors declare that the research was conducted in the absence of any commercial or financial relationships that could be construed as a potential conflict of interest.

## Publisher’s Note

All claims expressed in this article are solely those of the authors and do not necessarily represent those of their affiliated organizations, or those of the publisher, the editors and the reviewers. Any product that may be evaluated in this article, or claim that may be made by its manufacturer, is not guaranteed or endorsed by the publisher.
